# Mission and One-Year Experience of a Kidney–Heart Outpatient Service: A Patient-Centered Management Model

**DOI:** 10.3390/jcm14062102

**Published:** 2025-03-19

**Authors:** Caterina Carollo, Salvatore Evola, Alessandra Sorce, Emanuele Cirafici, Miriam Bennici, Giuseppe Mulè, Giulio Geraci

**Affiliations:** 1 Department of Health Promotion, Mother and Child Care, Internal Medicine and Medical Specialties (ProMISE) “G. D’Alessandro”, University of Palermo, 90133 Palermo, Italyemanuele.cirafici@community.unipa.it (E.C.); miriam.bennici@community.unipa.it (M.B.); giuseppe.mule@unipa.it (G.M.); 2 Catheterization Laboratory, Department of Medicine and Cardiology, Azienda Ospedaliera Universitaria Policlinico “P. Giaccone”, 90127 Palermo, Italy; salvatore.evola@policlinico.pa.it; 3 Department of Medicine and Surgery, “Kore” University of Enna, 94100 Enna, Italy; giulio.geraci@unikore.it

**Keywords:** cardiorenal, clinical nephrology, referral, consultation

## Abstract

**Background**: Cardiorenal Syndrome (CRS) represents a growing global health challenge due to the increasing prevalence of coexisting kidney and heart disease. The complex pathophysiology of CRS demands an integrated, multidisciplinary approach involving both nephrology and cardiology. However, specialized care models remain limited, leading to fragmented management and suboptimal outcomes. **Methods**: A Kidney–Heart Outpatient Service was established at "Paolo Giaccone" University Hospital in Palermo in May 2023 to provide coordinated, multidisciplinary care for non-hospitalized patients with CRS. The service involves structured patient assessments, including medical history, physical examinations, laboratory tests, imaging, and a collaborative therapeutic plan formulated by nephrologists and cardiologists. Preliminary patient data were collected and analysed to assess demographic characteristics, comorbidities, and clinical outcomes. **Results**: Among the first 115 patients evaluated, most were male and over 70 years old. Hypertension (91%) and diabetes were the leading comorbidities, with CKD stage G3b being the most prevalent. Cardiovascular conditions such as atrial fibrillation (18%), prior myocardial infarction (17%), and heart failure (15%) were frequently observed. Three patient deaths occurred, and one progressed to hemodialysis. **Conclusions**: The Kidney–Heart Outpatient Service represents a novel, patient-centered model for CRS management, aiming to improve clinical outcomes and reduce hospital admissions through multidisciplinary collaboration. Longitudinal follow-up and expanded data collection are essential to validate the long-term efficacy of this approach and refine management strategies for CRS patients. Ongoing research efforts will focus on tracking patient outcomes over extended periods, optimizing therapeutic strategies, and further integrating nephrology and cardiology training. The goal is to establish a sustainable and scalable framework for CRS management that enhances patient care and reduces the healthcare burden.

## 1. Cardiorenal Syndrome: A Multidisciplinary Challenge Requiring Integrated Care

The number of patients with, or at risk for, coexisting kidney and heart disease, often named “cardiorenal” or “renocardiac” syndromes (CRS), is increasing worldwide. A lot of studies have documented higher mortality and significant morbidity in this kind of patients [1,2].

There is a significant interplay between nephrology and cardiology across various aspects, including epidemiology, risk factors, pathophysiology, diagnosis, prognosis, prevention, treatment, monitoring, and research, all of which involve both the kidney and the heart in cardiorenal patients, particularly those with renocardiac syndromes.

The Acute Dialysis Quality Initiative outlined a consensus-based approach in 2008, which categorized cardiorenal syndromes (CRS) into two main groups—cardiorenal and renocardiac syndromes—based on the primum movens of the disease process [3]. This classification was further subdivided into five subtypes, considering the acuity of the disease and the sequential involvement of organs. A scientific statement from the American Heart Association has expanded on the central concept that in CRS, acute or chronic dysfunction in one organ can induce acute or chronic dysfunction in the other organ [4].

This interaction between kidney and heart disease is attracting attention to hemodynamic factors, physiochemical variables and biological processes, leading to a complex pathophysiology with typical disease features, and to the need for specific treatment and specialized care that may not be adequately addressed by either cardiologist or nephrologist alone [5].

To address these challenges, nephrologists must undergo specialized training to enhance their cardiological knowledge and skills, while cardiologists must similarly deepen their understanding of nephrological aspects. Both disciplines should collaborate in a multidisciplinary care setting to advance the subspecialty of cardionephrology and improve outcomes in cardiorenal care [6].

It thus clearly emerges that everywhere, independent of the care setting, there is an urgent, unsatisfied need to take care of patients with combined kidney and heart disease [7,8] with a personalized, patient-centered approach.

## 2. Paradigm Shifts in Patient Management and Institutional Challenges

Historically, therapeutic practices have evolved significantly, moving from a “patient-centered” approach to one based on “evidence-based medicine”. This shift reflects a transition from a “holistic perspective” of disease to a focus on “specific causes for specific diseases” [9,10].

These paradigm shifts impact not only patient management but also the beliefs, habits, technical skills, and managerial competencies of all healthcare professionals involved in CRM syndrome care. Beyond daily disease management, clinicians must actively engage in this shift to ensure effective long-term care.

A holistic, multidisciplinary approach is essential, considering patients as individuals with complex medical, social, economic, and cultural needs. Collaboration among specialists is crucial, extending beyond in-office consultations to digital interactions and proactive follow-up, facilitated by nursing support. Excellence in scientific expertise is no longer sufficient—proficiency in management and patient care strategies is equally vital.

Currently in Italy, as in many other countries, cardiologists primarily manage cardiorenal syndromes of types 1 and 2, while nephrologists primarily treat types 3 and 4. However, there is a lack of synergy in efforts for the prevention and management of CRS at various levels. Institutional collaboration is minimal, often occurring only occasionally through consultations, which frequently take place too late in the patient care process. An integrated, simultaneous multidisciplinary approach involving the relevant healthcare professionals in CRS management is still lacking, leaving patients to receive partial or restricted care due to the narrow perspective of the primary specialty and limited resources in an outpatient setting [11].

We hypothesize that a shift in a similar paradigm may occur in nephrology and cardiology, particularly regarding patient stratification and targeted management.

Moreover, in addition to new drugs, recent advances in medical technologies and devices could offer the opportunity to better treat these patients [12,13].

## 3. Epidemiological Insights in Cardiorenal Syndrome

Cardiovascular diseases (CVD) are a leading cause of mortality in patients with chronic kidney disease (CKD), particularly in stage 5 (dialysis-dependent), where they account for up to 58% of deaths [1,14]. In addition to traditional cardiovascular risk factors (CVRFs) such as diabetes and hypertension, non-traditional risk factors associated with kidney disease itself appear to play a crucial role in the complex interaction between the kidney and the heart. CKD is prevalent among HF patients and significantly contributes to increased mortality rates. Data from 1999 to 2020 indicate that cardiovascular diseases accounted for 31.2% of deaths in patients with CKD [15]. A meta-analysis of over 3.5 million heart failure patients estimated the incidence of acute kidney injury (AKI) at approximately 33% [16]. Among those hospitalized with acute decompensated heart failure (ADHF), the prevalence of AKI is around 20% [17]. The development of AKI in heart failure patients is associated with a significant increase in in-hospital mortality, with affected individuals exhibiting a higher likelihood of adverse outcomes compared to those without AKI [18]. In the context of acute decompensated heart failure complicated by cardiogenic shock (ADHF-CS), AKI serves as an independent predictor of 90-day mortality [19].

**Figure 1:**
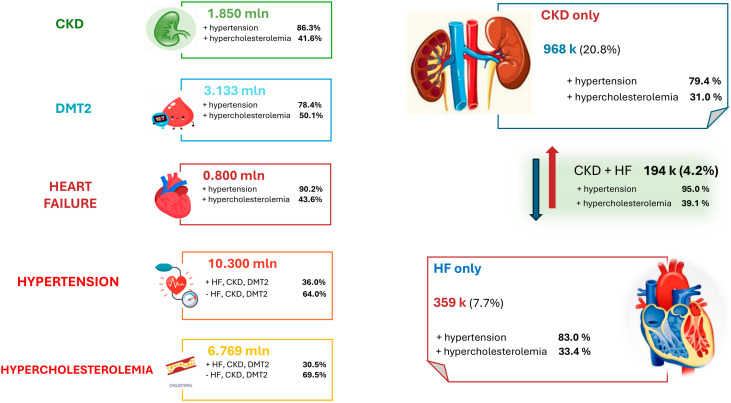
Epidemiological impact of cardiorenal metabolic syndrome. Sources: Deloitte reanalysis of data from the Clicon CRM Observatory (2023) [20].

A reanalysis conducted by Deloitte using data from the Clicon CRM Observatory (2023) highlights a progressive increase in the incidence of cardiometabolic risk factors as chronic kidney disease (CKD) advances. In patients with stage 3B CKD, the prevalence of type 2 diabetes and heart failure is 28% and 73% higher, respectively, compared to those in stage 3A. The transition to stage 4 CKD is associated with a further increase of 10% in type 2 diabetes and 37% in heart failure prevalence. Additionally, an analysis integrating data from the Clicon CRM Observatory with epidemiological findings from leading scientific societies in cardiology, diabetology, and nephrology estimates that approximately 11.528 million individuals in Italy have been diagnosed with cardiorenal-metabolic syndrome [20]. Regarding cardiorenal syndrome, the incidence data for Italy are illustrated in Figure 1.

Cardiorenal metabolic syndrome (CRM) represents a significant challenge for the National Health Service (SSN), not only due to its prevalence but also because of the high degree of clinical complexity and patient frailty it entails. Frailty is defined by clinical complexity, which is determined by the number and severity of comorbid conditions, biological susceptibility, social vulnerability, and the organizational challenges associated with patient management [21].

The reanalysis of data from the Clicon CRM Observatory (2023) by Deloitte indicates that CRM patients are often characterized by advanced age, with a mean age exceeding 73 years, and 24% being over 80 years old. They exhibit a high burden of comorbidities, with an average of 2.5 metabolic, cardiovascular, and renal risk factors, increasing to at least three in nearly half of the cases. Additionally, these patients demonstrate substantial disease severity, as evidenced by a hospitalization rate of approximately 26%, and a notable prevalence of depression, affecting 14.4% of cases.

## 4. Hospitalization Trends and Economic Burden

Due to their clinical complexity, CRS patients require frequent healthcare interventions, leading to significant resource utilization within the SSN: on an annual basis, these patients receive over 20 prescriptions, undergo specialist or diagnostic consultations approximately every six weeks, visit their general practitioner every 20 days, and experience hospitalization approximately once every two years.

The challenges associated with managing CRS patients extend beyond the interplay of multiple comorbidities, the presence of organ damage, and the critical importance of lifestyle modifications. Social and cultural factors further complicate their care, while the substantial clinical complexity inherent to these patients is both a cause and a consequence of the significant organizational burden they impose on the healthcare system.

Admissions and readmissions for decompensated heart failure are among the most common causes for hospitalizations in Italy; therefore, reducing frequency and improving outcomes associated with heart failure hospitalizations is a public health priority [22,23]. AKI frequently complicates these hospitalizations and is associated with poor clinical outcomes and higher resources utilization [24].

Preliminary findings on the impact of cardiorenal units on the clinical outcomes of patients with AKI and HF are encouraging, but further validation in larger patient populations and extended follow-up periods are necessary.

Cardiorenal units for inpatients provide support to nephrologists and cardiologists in various clinical settings, including regular medical wards, telemetry units, and intensive care units. These units aim to facilitate more consistent interdisciplinary communication, creating a clinical and educational environment that enhances the expertise of both specialties in managing cardiorenal conditions.

Furthermore, cardiorenal units play a critical role in advancing research on cardiorenal syndrome (CRS) and renocardiac syndromes. Key scientific components of these units include the longitudinal collection of electronic health records from cardiorenal patients and their direct recruitment into clinical research studies. It is essential to recognize the existing gap in evidence-based therapies for patients with chronic cardiorenal syndrome, particularly those with advanced CKD and chronic heart failure [25].

However, these data pertain to a management context related to hospitalizations, and the experiences of outpatient management for patients requiring such a combined approach are unfortunately rarely shared in the literature.

Effectively addressing CRS requires an optimized organizational framework that places patients at the center of healthcare efforts. A key challenge is the high proportion of undiagnosed cases, estimated at 40% of those currently in treatment [20]. Early detection, rather than diagnosis at advanced stages or after adverse events, is essential to slowing disease progression.

Robust primary prevention strategies are crucial. Individuals at risk—such as those with hypertension, hypercholesterolemia, obesity, advanced age, or a family history of CRS—should be engaged in systematic screening programs to enable early diagnosis.

Another challenge is the suboptimal appropriateness of treatment, compounded by poor therapeutic adherence and limited compliance with medical follow-ups. Structural inefficiencies in the public healthcare system have led to increased reliance on private healthcare, with 29% of Italians forgoing care due to access barriers and 35% paying out-of-pocket for essential services [26].

The lack of an integrated approach to CRS management exacerbates clinical complexity, increasing therapeutic inertia and poor adherence. The economic burden on the National Health Service (SSN) is significant, with cardiovascular disease costs reaching EUR 21.34 billion, 59% of which is attributed to hospital care [27]. A coordinated, multidisciplinary approach is essential to improving patient outcomes and reducing healthcare system strain.

## 5. The Kidney–Heart Outpatient Service: Our Experience

For all of these reasons, in May 2023 we launched the Kidney–Heart Outpatient Service in the Nephrology and Dialysis Unit of “Paolo Giaccone” University Hospital in Palermo, and to the best of our knowledge this is the first outpatient facility dedicated to nephrocardiology patients to have been established in our country.

The clinical mission of our Kidney–Heart Service is to provide coordinated, multi-disciplinary care for non-hospitalized patients with coexisting kidney and heart disease, with the aim of significantly reducing admissions and readmissions in hospital and improving patient outcomes and optimizing the utilization of economic resources.

To achieve this goal, the Service offers consultations for patients scheduled every week from a reservation list, which is created based on the prescriptions provided by general practitioners. The general practitioners identify the clinical criteria required by the definition of cardio-renal syndrome to classify these patients as cardio-renal, ensuring that patients who meet the necessary diagnostic thresholds are referred for specialized care. This process helps prioritize patients with complex, interrelated cardiovascular and renal issues, ensuring they receive the appropriate attention and management.

This Service has been staffed with a nephrologist and a cardiologist trained in point-of-care ultrasounds and it works once a week with five patients a time.

In a typical appointment, patients were asked about their medical history, then underwent to the medical examination: First, body weight, height, waist-to-hip ratio, and arterial pressure (expressed as a mean of three measurements) were collected. After that, a typical 12-derivation ECG was registered and biochemical exams were collected or prescribed. On this basis, further instrumental blood and urine exams were proposed: 24 h urine collection is routinary prescribed to enable protein excretion and microalbuminuria, along with blood lipid profile.

To better assess the cardio-renal damage, Fundus Oculi was obtained by a trained ophtalmologist from every patient with a portable ophtalmoscope. If necessary and urgent, lung, heart and kidney ultrasound scanning was obtained on the same occasion; otherwise, it was scheduled along with the carotid Doppler.

Finally, the nephrologist and cardiologist finish the evaluation and give the medical report to the patient. In the report, a complete, shared medical therapy is prescribed, according to the most recent guidelines.

**Figure 2:**
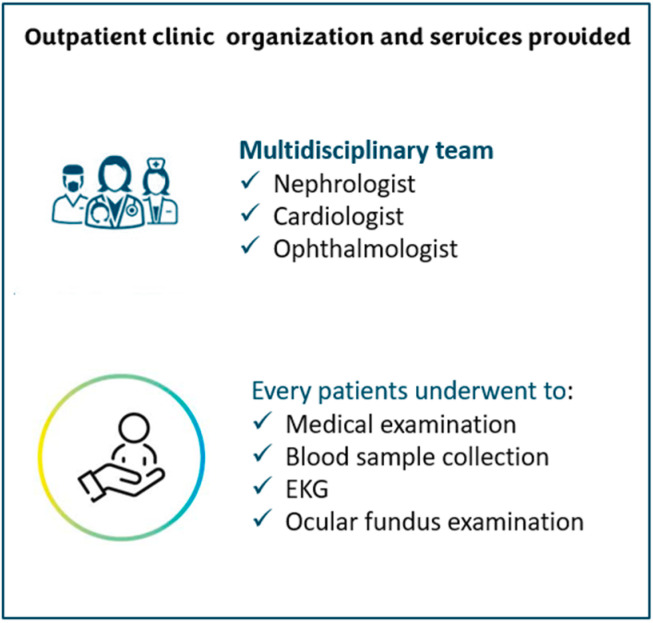
Outpatient clinic organization and services provided.

The information regarding the organization of the outpatient clinic and the services provided is illustrated in Figure 2.

The most important objectives are the reaching of blood pressure goals and the normalization of blood glucose levels and the lipid profile; therefore, diet and lifestyle modifications, along with drugs, are prescribed. Drug dosages are obviously adjusted for eGFR obtained by CKD-EPI equation.

A combined-care model involving specialists in cardiology and nephrology could substantially redefine the management of pharmacological therapies commonly used in patients with cardiorenal syndrome. Specifically, it could lead to a more integrated approach in the use of inotropic agents, vasopressors, diuretics, and renin-angiotensin system inhibitors, as well as with anticoagulants, lipid-lowering agents, and medications to modify electrolytes.

## 6. Preliminary Data

**Figure 3:**
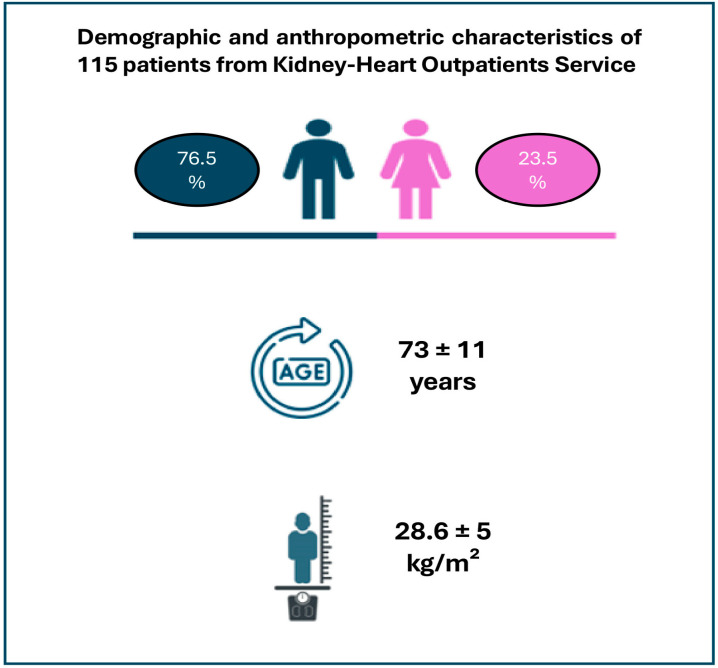
Demographic and anthropometric characteristic of 115 patients from the Kidney–Heart Outpatient Service.

In the following figure, we summarize the main features of the patients we evaluated from the launch of the Kidney–Heart Outpatient Service. As can be seen, most of the patients were male and aged over 70 y (Figure 3).

Hypertension and diabetes confirm their leading role both in inducing and accompanying kidney disease, along with myocardial infarction and atrial fibrillation.

Regarding the CKD Stage, G3b is the most frequent observed.

**Figure 4:**
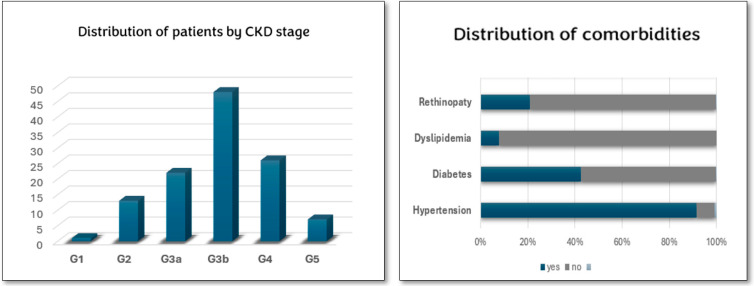
Distribution of main comorbidities and CKD stage in our cohort. (Stages of chronic kidney disease (CKD) classified based on glomerular filtration rate (GFR). **G1:** Normal or high kidney function (GFR ≥ 90 mL/min/1.73 m²) with kidney damage. **G2:** Mildly decreased function (GFR 60–89 mL/min/1.73 m²) with kidney damage. **G3a:** Mild to moderate reduction in function (GFR 45–59 mL/min/1.73 m²). **G3b:** Moderate to severe reduction (GFR 30–44 mL/min/1.73 m²). **G4:** Severe reduction (GFR 15–29 mL/min/1.73 m²). **G5:** Kidney failure (GFR < 15 mL/min/1.73 m²), requiring dialysis or transplantation).

We found a high prevalence of arterial hypertension (91%) in our patients, the relative details of which are illustrated in the image below (Figure 4).

**Figure 5:**
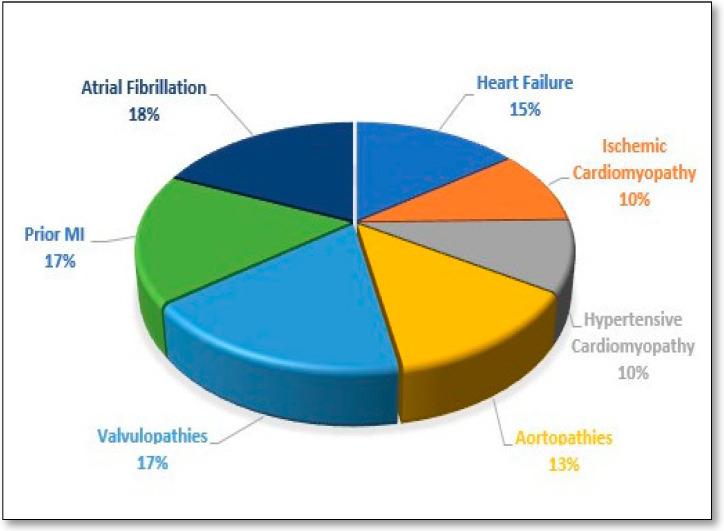
Distribution of cardiovascular diseases in our cohort.

The pie chart (Figure 5) illustrates the distribution of cardiovascular diseases, highlighting that Atrial Fibrillation accounts for 18%, Prior AMI for 17%, Valvulopathies for 17%, and HF FOR 15% (as the highest percentages).

We registered three deaths (one for stroke, one for acute heart failure, and the last for myocardial infarction). One of our patients underwent hemodialysis.

To fulfill the requirements of a University Hospital, we involved postgraduate medical students from both Nephrology and Cardiology postgraduate medical schools in the decision-making process. Since our goal is to encourage the acquisition of multidisciplinary knowledge and to prevent trainees from inheriting the outdated view of a clear separation between their respective areas of interest, we aim to ensure that future specialists do not enter into conflict or provide only partial consultations and therapeutic strategies with a limited perspective, to the detriment of productive cooperation. Internal Medicine postgraduate medical students were also welcome.

## 7. Future Directions

Our early preliminary data need to be confirmed over a longer period after the launch of the Kidney–Heart Outpatient Service. We have remained committed to our research objectives, which is why we are actively collecting and analyzing longitudinal medical record data from patients who have been seen by the Kidney–Heart Service. Our goal is to closely track the long-term progression of these patients, not only during their hospitalizations but also throughout their outpatient follow-ups. By monitoring their health outcomes both in and out of the hospital, we aim to gain a deeper understanding of the effectiveness of combined nephrocardiology treatments, identify potential gaps in care, and ultimately improve patient management strategies for individuals with complex cardiovascular and renal conditions. This longitudinal approach will allow us to assess trends, recognize early signs of complications, and refine treatment protocols to enhance patient care across both hospital and outpatient settings.

## Data Availability

No new data were created or analyzed in this study.
